# Longitudinal Analysis of Anti-cardiolipin and Anti-β2-glycoprotein-I Antibodies in Recent-Onset Systemic Lupus Erythematosus: A Prospective Study in Swedish Patients

**DOI:** 10.3389/fmed.2021.646846

**Published:** 2021-02-24

**Authors:** Martina Frodlund, Tomas Walhelm, Charlotte Dahle, Christopher Sjöwall

**Affiliations:** ^1^Division of Inflammation and Infection/Rheumatology, Department of Biomedical and Clinical Sciences, Linköping University, Linköping, Sweden; ^2^Division of Inflammation and Infection/Clinical Immunology and Transfusion Medicine, Department of Biomedical and Clinical Sciences, Linköping University, Linköping, Sweden

**Keywords:** anti-phospholipid antibodies, anti-cardiolipin antibodies, anti-β2-glycoprotein-I antibodies, cardiovascular events, longitudinal analysis, pregnancy morbidities, systemic lupus erthematosus, antiphospholipid antibody syndrome

## Abstract

**Background:** Anti-phospholipid syndrome (APS) and systemic lupus erythematous (SLE) are autoimmune disorders that often co-occur. Anti-phospholipid antibodies (aPL) are typical of both conditions and may be associated with vascular events and pregnancy-related morbidities. Whereas, aPL-screening is mandatory for individuals with suspected SLE, the clinical value of longitudinal aPL analyses in established SLE is unclear.

**Methods:** We investigated the occurrence and variation of IgG/IgA/IgM anti-cardiolipin (aCL) and anti-β2-glycoprotein-I (anti-β2GPI) antibodies, using both the manufacturer's cut-off and a cut-off based on the 99th percentile of 400 apparently healthy donors, in recent-onset SLE. Furthermore, we evaluated the relationships between aPL levels and SLE/APS manifestations, as well as the pharmacotherapy. Patients with SLE who met validated classification criteria were included in this prospective study (*N* = 54). Samples were obtained at 0, 6, 12, 24, 36, 48, 60, 72, 84, and 96 months after SLE diagnosis.

**Results:** Depending on the cut-off applied, 61.1 or 44.4% showed a positive result for at least one aPL isotype or the lupus anticoagulant test over time. Median values for all six aPL isotypes numerically decreased from inclusion to last follow-up, but none of the isotypes met statistical significance. Seroconversion (from positive to negative, or the opposite direction) was occasionally seen for both aCL and anti-β2GPI. IgA and IgM anti-β2GPI were the most common isotypes, followed by IgM aCL. Presence of IgG aCL associated significantly with myocardial infarction and miscarriage, and IgG/IgA anti-β2GPI with miscarriage.

**Conclusion:** aPL were common during the first years of SLE. Even though the levels fluctuated over time, the patients tended to remain aPL positive or negative. Repeated aPL testing in the absence of new symptoms seems to be of uncertain value in patients with recent-onset SLE.

## Introduction

Although antiphospholipid syndrome (APS) and systemic lupus erythematous (SLE) are distinct autoimmune disorders, they share several features and are often regarded as two sides of the same coin (1, 2). Indeed, the clinical presentations of Libman-Sacks endocarditis, livedo reticularis, migraine or autoimmune cytopenia, together with laboratory findings in relation to complement consumption, antinuclear and antiphospholipid antibodies (aPL), are commonly seen in both conditions (3). Regardless of whether APS appears on its own or concomitantly with (secondary to) other autoimmune conditions, it is classified based on the presence of one or more repeated aPL tests and at least one of two major clinical manifestations, thrombosis or pregnancy complications.

The validated APS classification criteria from 2006 include immunoglobulin (Ig)G/IgM anti-cardiolipin antibodies (aCL), IgG/IgM anti-β2-glycoprotein-I antibodies (anti-β2GPI), and the lupus anticoagulant (LA) test (4). The percentage of subjects with SLE who occasionally or continuously test positive for aPL has been estimated as in the range of 30–40%, and about half of these patients eventually fulfill the APS criteria (5–7).

There has been intense discussion regarding “seronegative” APS, since some patients with a clinical APS phenotype show negative results for the classical aPL tests (8). Indeed, antibodies directed against other proteins of the coagulation cascade, such as prothrombin and phosphatidylserine-prothrombin complexes, are examples of other aPL that are not (yet) included in APS classification criteria (1). This is because they are not used routinely, or there is some uncertainty as to their clinical significance, or there is a lack of standardized testing. Some studies have shown that one of the five domains of β2GPI, domain I, is of particular importance for the pathogenesis of APS (9, 10). Antibodies directed against this specific domain appear to be strongly associated with thrombosis, as well as with obstetric complications, as compared to antibodies that target the entire β2GPI molecule (11).

In SLE, the presence of aPL has been associated with thrombosis and/or pregnancy morbidities, as well as with worse prognosis involving increased damage accrual (12–15). A positive LA test has been identified as the single laboratory finding with the highest predictive value regarding future organ damage in patients with SLE (16). In contrast to the 2006 APS classification criteria and older SLE criteria sets, the 2012 Systemic Lupus International Collaborating Clinics (SLICC) and the 2019 European League Against Rheumatism (EULAR)/American College of Rheumatology (ACR) classification criteria include the IgA isotype for aCL and anti-β2GPI (2, 17). Some reports have suggested that the IgA isotype has additional value for the risk assessment of thrombosis and pregnancy morbidities (18–20). Other data indicate that the significance of IgA aPL varies across different ethnicities (21, 22). Our own recent study based on a Swedish (mainly Caucasian) SLE population concluded that IgA aPL alone are of additional value only for patients with a high probability of APS, despite negative test outcomes for IgG/IgM aCL, anti-β2GPI and LA (23). Similar data were recently reported from a large Chinese population (24).

Whereas, screening for aPL is mandatory during the investigation of individuals with suspected recent-onset APS or SLE, the clinical value of longitudinal aPL analyses in those patients with an established SLE diagnosis remains uncertain. Already in 1989, fluctuations of aCL levels over time in subjects with SLE were reported, and the variability of anti-β2GPI levels was also highlighted (25, 26). However, data regarding longitudinal prospective studies of aPL in patients with recent-onset SLE are limited (27). In primary APS, <10% of patients switched from being aPL positive to negative over 5 years of follow-up (28).

The primary aim of the present study was to investigate the presence and fluctuation of aPL isotype (IgG, IgA, and IgM) levels in patients with recent-onset SLE. The secondary aims were to evaluate the potential relationships between aPL levels and smoking habits, pharmacotherapy, SLE and APS manifestations, clinical and laboratory markers of disease activity, and organ damage accrual.

## Materials and Methods

### Subjects

The 54 patients with newly diagnosed SLE (≤ 6 months of symptoms) included in this study have been described previously (29). All the subjects met the 1982 ACR criteria and/or the 2012 SLICC criteria and had taken part in the prospective follow-up program KLURING (Swedish acronym for Clinical LUpus Register In Northeastern Gothia) at the Rheumatology Clinic, Linköping University Hospital (30). APS was classified according to the Sydney criteria (4). Clinical follow-up data and serum samples (stored at −70°C) were collected from the time of SLE diagnosis (Month 0) and thereafter, in most cases yearly.

Pharmacotherapy [azathioprine, belimumab, cyclophosphamide, hydroxychloroquine (HCQ), methotrexate, mycophenolate mofetil, rituximab, prednisolone and sirolimus] was recorded at each visit. The daily dose of prednisolone was calculated as a continuous variable. SLE disease activity was assessed using the clinical SLE disease activity index 2000 (cSLEDAI-2K) (which excludes items for low complement levels and positive anti-dsDNA) (31). Organ damage, required to have been persistent for ≥6 months, was recorded annually by the SLICC/ACR damage index (SDI), which encompasses damage in 12 defined organ systems (32). A detailed description of the study population is given in Table 1.

**Table 1:** Characteristics of the included patients with recent-onset SLE (*N* = 54).

	**At inclusion**	**At last follow-up**
**Background variables**		
Age, mean (range), years	44 (18–82)	
Gender, female, *N* (%)	45 (83.3)	
Ever smoker (former or current), *N* (%)	23 (42.6)	
Body mass index, mean (range)	25.1 (17.8–40.4)	
Caucasian ethnicity, *N* (%)	51 (94.4)	
**Clinical APS phenotypes**, ***N*** **(%)**		
APS (clinical diagnosis)	8 (14.8)	
APS (defined by classification^*^)	7 (13)	
cSLEDAI at inclusion, mean (range)	2.6 (0–20)	0.4 (0–8)
SDI at last visit, mean (range)	N/A	0.8 (0–5)
Low complement (%)	24 (44.4)	15 (27.8)
Meeting SLICC-12 criteria, *N* (%)	52 (96.3)	
Meeting ACR-82 criteria, *N* (%)	44 (82.0)	47 (87.0)
Number of fulfilled ACR-82 criteria, mean (range)	4.4 (3–9)	4.6 (3–9)
**Clinical SLE phenotypes (ACR-82 definitions)**, ***N*** **(%)**		
Malar rash	17 (31.5)	17 (31.5)
Discoid lupus	6 (11.1)	6 (11.1)
Photosensitivity	30 (55.6)	30 (55.6)
Oral ulcers	8 (14.8)	9 (16.7)
Arthritis	41 (75.9)	41 (75.9)
Serositis	18 (33.3)	19 (35.2)
Renal disorder	6 (11.1)	8 (14.8)
Neurological disorder	1 (1.9)	2 (3.7)
Hematological disorder	25 (46.3)	30 (55.6)
Immunological disorder	27 (50.0)	30 (55.6)
Anti-nuclear antibody^#^	54 (100)	54 (100)
**Immunomodulatory therapies**, ***N*** **(%)**		
Azathioprine	4 (7.4)	3 (5.6)
Belimumab	0	1 (1.9)
Cyclophosphamide	1 (1.9)	0
Hydroxychloroquine	41 (74.1)	41 (74.1)
Methotrexate	7 (13.0)	4 (7.4)
Mycophenolate mofetil	0	3 (5.6)
Prednisolone, median dose (range) in mg	5 (0–60)	5 (0–30)
Rituximab	1 (1.9)	0
Sirolimus	0	1 (1.9)

* *According to Sydney criteria (4)*.

# *Positive by immunofluorescence microscopy (IF-ANA)*.

*ACR, American College of Rheumatology; APS, anti-phospholipid syndrome; cSLEDAI, clinical SLE disease activity 2000 (SLEDAI-2K) score; MI, myocardial infarction; N/A, not applicable; SDI, SLICC/ACR damage index; SLICC, systemic lupus international collaborating clinics*.

### aPL Assays

Sera for the detection of IgG/IgA/IgM aCL and anti-β2GPI were available from the time-points of 0, 6, 12, 24, 36, 48, 60, 72, 84, and 96 months after the diagnosis of SLE. The analyses were performed by the personnel at the accredited Clinical Immunology laboratory at Linköping University Hospital using a fluoroenzyme-immunoassay (Phadia-250 instrument; Thermo-Fisher Scientific Phadia AB, Uppsala, Sweden). The cut-offs for each aPL isotype were set according to the manufacturer's instructions; for IgG and IgM aCL, positive results were defined as ≥10 U/mL and for IgA aCL ≥14 U/mL. For IgG, IgA and IgM anti-β2GPI, positive results were defined as ≥7 U/mL. Outcomes below the cut-off for each antibody were ascribed half the cut-off value in the statistical analyses. To comply with the Sydney criteria of APS, cut-offs based on the 99th percentile of apparently healthy donors (*N* = 400; 50% males, 50% females) were set and positive results were defined as follows: IgG aCL ≥24 U/mL, IgA aCL ≥17 U/mL, IgM aCL ≥30 U/mL, IgG anti-β2GPI ≥18 U/mL, IgA anti-β2GPI ≥9 U/mL, and IgM anti-β2GPI ≥6 U/mL (4). All samples were analyzed at the same occasion to minimize inter-assay variation.

The results of the LA tests, which were performed using the dilute Russell's viper venom time (dRVVT) method at the Clinical Chemistry laboratory at Linköping University Hospital, were retrieved from the medical records.

### Statistics

For comparisons of aPL levels between groups, the Mann-Whitney *U*-test was used. Associations between aPL positivity (categorical variable) and APS-related events, SLE manifestations and organ damage were examined with the χ^2^-test, or Fisher's exact-test when appropriate (*N* ≤ 5). *P*-values ≤ 0.05 were considered statistically significant. Statistical analyses were performed using the SPSS software ver. 26.0.0.0 (SPSS Inc., Chicago, IL, USA) or GraphPad Prism ver. 8.4.3 (GraphPad Software Inc., San Diego, CA). Graphs were created using GraphPad Prism ver.8.4.3 (GraphPad Software).

### Ethics Statement

Oral and written informed consent was obtained from all the participants. The study protocol was approved by the Regional Ethics Review Board in Linköping (Decision Nr. M75-08/2008).

## Results

### Prevalence of aPL

Table 2 lists the prevalence rates of aPL among the participating subjects at inclusion and longitudinally using the manufacturer's cut-offs or the more stringent 99th percentile cut-offs shown in parenthesis. Overall, 38.9% (24.1%) were ever positive for any aCL isotype. Regarding anti-β2GPI, 42.6% (37.0%) of the 54 patients showed ever positivity for any anti-β2GPI isotype. A positive LA test was recorded for 16 cases (29.6%). LA was initially controlled in all patients adjacent to the time-point of SLE diagnosis as part of clinical routine. In all patients with a positive LA test, the test was confirmed positive with a new sample and use of the same assay (dRVVT).

**Table 2:** Frequencies of aPL using either the manufacturer's or the 99th percentile's cut-off by each antiphospholipid antibody isotype in the included patients with recent-onset SLE.

	**At inclusion, manufacturer's cut-off, *N* (%)**	**Ever positive, manufacturer's cut-off, *N* (%)**	**At inclusion, 99^TH^ percentile cut-off, *N* (%)**	**Ever positive, 99^TH^ percentile cut-off, *N* (%)**
**aCL isotypes**				
Any aCL	15 (27.8)	21 (38.9)	10 (18.5)	13 (24.1)
IgG	7 (13.0)	11 (20.4)	5 (9.3)	7 (13.0)
IgA	4 (7.4)	4 (7.4)	3 (5.6)	3 (5.6)
IgM	11 (20.4)	14 (25.9)	5 (9.3)	8 (14.8)
IgG, IgA	2 (3.7)	2 (3.7)	2 (3.7)	2 (3.7)
IgG, IgM	4 (7.4)	5 (9.3)	2 (3.7)	3 (5.6)
IgM, IgA	2 (3.7)	2 (3.7)	1 (1.9)	1 (1.9)
IgG, IgM, IgA	1 (1.9)	1 (1.9)	1 (1.9)	1 (1.9)
**anti-β2GPI isotypes**				
Any anti-β2GPI	17 (31.5)	23 (42.6)	16 (29.6)	20 (37.0)
IgG	8 (14.8)	9 (16.7)	3 (5.6)	5 (9.3)
IgA	14 (25.9)	16 (29.6)	11 (20.4)	12 (22.2)
IgM	8 (14.8)	12 (22.2)	9 (16.7)	13 (24.1)
IgG, IgA	4 (7.4)	6 (11.1)	1 (1.9)	2 (3.7)
IgG, IgM	3 (5.5)	5 (9.3)	2 (3.7)	3 (5.6)
IgM, IgA	7 (13.0)	8 (14.8)	6 (11.1)	7 (13.0)
IgG, IgM, IgA	3 (5.5)	5 (9.3)	2 (3.7)	2 (3.7)
**LA**	N/A	16 (29.6)	N/A	16 (29.6)
Triple-positive^*^	N/A	8 (14.8)	N/A	8 (14.8)

*N/A, Not applicable; LA, Lupus anticoagulant test*.

* *Positive for any isotype of aCL, any isotype of anti-β2GPI combined with positive lupus anticoagulant test*.

### Longitudinal aPL Analyses and Seroconversion

**Figure 1:**
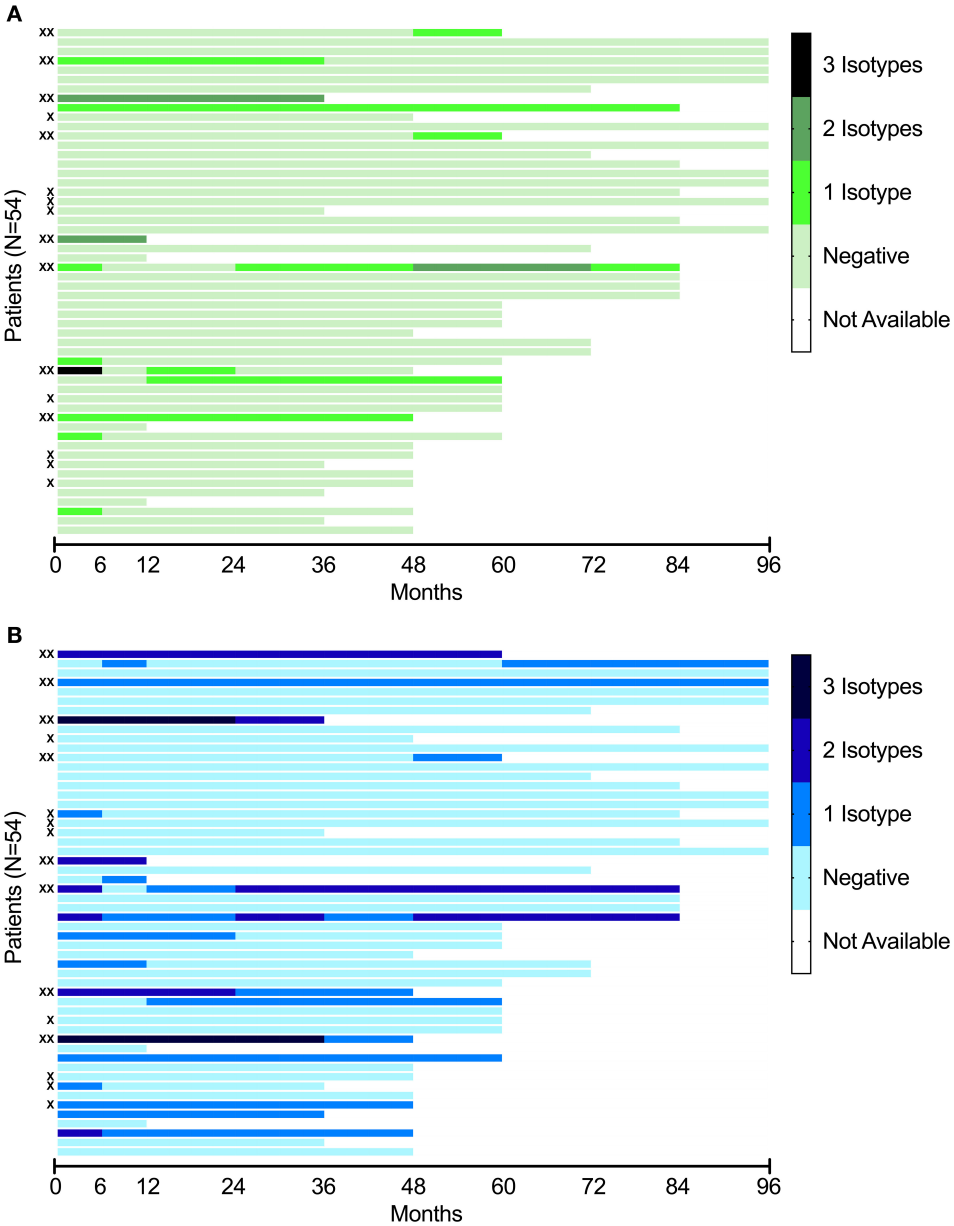
**(A)** Longitudinal analysis of aCL of three isotypes (IgG, IgA, IgM) in the included patients with recent-onset SLE (*N* = 54). Each line represents a single patient, in the same order as in this figure **(B)**. **(B)** Longitudinal analysis of anti-β2GPI of three isotypes (IgG, IgA, IgM) in the included patients with recent-onset SLE (*N* = 54). Each line represents a single patient, in the same order as in this figure **(A)**. **X** indicate cases with a positive LA test. **XX** indicate triple-positive subjects (individuals positive for any isotype of aCL, any isotype of anti-β2GPI combined with a positive LA test).

Longitudinal data for the positive aPL isotypes for aCL and anti-β2GPI, separately, are illustrated in Figure 1. Complete data for 50/54 patients (92.6%) were available up to 36 months from the time of SLE diagnosis. By comparing the inclusion sample with the last follow-up, four cases converted from positive aCL to negative and three patients showed the opposite conversion (from negative to positive) (Figure 1A). Regarding anti-β2GPI, four subjects converted from positive to negative and four patients initially showed positive anti-β2GPI at inclusion but were negative at last follow-up (Figure 1B). Median values for all six aPL isotypes numerically decreased from inclusion to last follow-up, but none met statistical significance. IgG anti-β2GPI were borderline significant (*p* = 0.068).

Seven patients tested positive for IgG aCL at inclusion (five patients using the 99th percentile cut-off) and five were still positive at the last follow-up visit (three patients using the 99th percentile cut-off). Only one of the four IgA aCL-positive cases at inclusion was persistently positive, regardless of which cut-off that was used. Of the 11 cases that were IgM aCL-positive at inclusion (five patients using the 99th percentile cut-off), six were still positive at the last follow-up visit (one patient using the 99th percentile cut-off). Initially, 72.2% (83.3% using the 99th percentile cut-offs) were aCL-negative and 61.1% (85.2% using the 99th percentile cut-off) were still negative at the last follow-up.

Of the eight IgG anti-β2GPI-positive cases at inclusion (three patients using the 99th percentile cut-off), only two (one patient using the 99th percentile cut-off) were persistently positive. Regarding the IgA anti-β2GPI-positive subjects, ten out of 14 (seven patients of 11 using the 99th percentile cut-off) were persistently positive. Of the eight IgM anti-β2GPI-positive cases at inclusion (nine patients using the 99th percentile cut-off), three were persistently positive at the last follow-up visit regardless of cut-off applied. Initially, 68.5% (72.2% using the 99th percentile cut-off) were anti-β2GPI-negative and 31 subjects were still negative at the last follow-up (34 using the 99th percentile cut-off).

**Figure 2:**
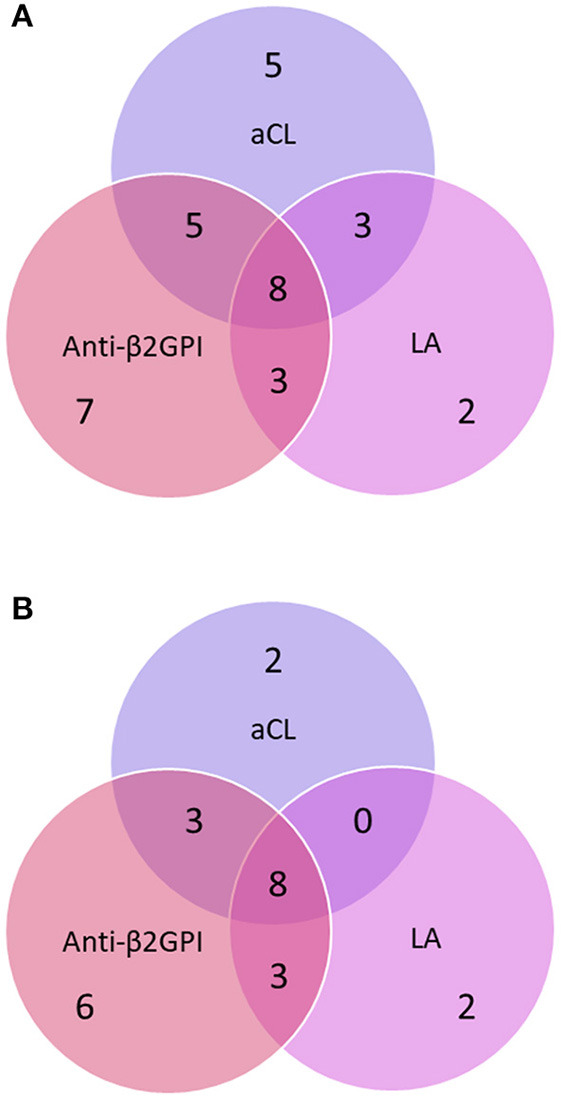
**(A)** Distribution of anti-phospholipid antibodies (aPL) using the manufacturer's cut-offs among patients who tested positive on at least one occasion during the study period. **(B)** Distribution of anti-phospholipid antibodies (aPL) using the 99th percentile cut-offs among patients who tested positive on at least one occasion during the study period. The circles represent IgG/IgA/IgM anti-cardiolipin antibody (aCL), anti-β2-glycoprotein-I (anti-β2GPI) and the lupus anticoagulant (LA) test.

Over time, 33 (61.1%) out of 54 patients showed a positive result for at least one aPL isotype or the LA test [24 patients using the 99th percentile cut-offs (44.4%)]. The distributions of the aPL-positive cases are illustrated in Venn diagrams with the different cut-offs applied (Figure 2).

### Longitudinal aPL and Clinical Outcome

Table 3 shows detailed information on aPL positivity (manufacturer's cut-off applied) and the vascular events experienced by the included patients during the study period. Ever positivity for IgG aCL as a categorical variable was significantly associated with any vascular event [i.e., cerebrovascular lesions, transient ischemic attack, myocardial infarction (MI), pulmonary embolism and deep vein thrombosis], as compared with the IgG aCL-negative cases (*p* <0.002).

**Table 3:** Vascular events vs. antiphospholipid antibodies (manufacturer's cut-off applied) in the included patients with recent-onset SLE during the study period (*N* = 54).

**Type of VE**	***N*, %**	aPL negative^*^ (*N* = 21)	**aCL (*****N*** **=** **21)**			**Anti-β2GPI (*****N*** **=** **23)**			**LA (*N* = 16)**	**Triple-positive^#^ (*N* = 8)**
			**IgG**	**IgA**	**IgM**	**IgG**	**IgA**	**IgM**		
All VEs	13 (24.1)	3 (23)	6 (46)	0 (0)	3 (23)	4 (31)	3 (23)	4 (31)	6 (46)	2 (15)
Any arterial event	4 (7.4)	1 (25)	2 (50)	0 (0)	1 (25)	1 (25)	0 (0)	0 (0)	2 (50)	1 (25)
CVL	4 (7.4)	1 (25)	2 (50)	0 (0)	1 (25)	1 (25)	0 (0)	0 (0)	2 (50)	1 (25)
Ischemic stroke	3 (5.6)	1 (33)	2 (67)	0 (0)	1 (33)	1 (33)	0 (0)	0 (0)	1 (33)	1 (33)
TIA	3 (5.6)	0 (0)	1 (33)	0 (0)	0 (0)	1 (33)	0 (0)	0 (0)	2 (67)	0 (0)
MI	2 (3.7)	0 (0)	2 (100)	0 (0)	1 (50)	1 (50)	0 (0)	0 (0)	1 (50)	1 (50)
Any venous event	5 (9.3)	1 (20)	2 (40)	0 (0)	2 (40)	1 (20)	1 (20)	3 (60)	1 (20)	1 (20)
PE	3 (5.6)	0 (0)	1 (33)	0 (0)	1 (33)	0 (0)	1 (33)	2 (67)	1 (33)	0 (0)
DVT	2 (3.7)	1 (50)	1 (50)	0 (0)	1 (50)	1 (50)	1 (50)	1 (50)	1 (50)	1 (50)
Late miscarriage (at or after gestational week 10)	2 (4.4)	0 (0)	2 (100)	0 (0)	1 (50)	2 (100)	2 (100)	2 (100)	2 (100)	2 (100)

* *Continuously negative for aCL, anti-β2GPI and LA*.

# *Positive for any isotype of aCL and any isotype of anti-β2GPI, combined with a positive lupus anticoagulant test*.

*CVL, cerebrovascular lesion; DVT, deep vein thrombosis; MI, myocardial infarction; PE, pulmonary embolism; TIA, transient ischemic attack; VE, vascular event*.

The median longitudinal IgG aCL levels were significantly higher among cases with MI compared with cases without MI (55 vs. 11 U/mL; *p* < 0.0001) (Supplementary Figure 1A). Females who reported late miscarriage (≥10th week of pregnancy) had significantly higher median levels of IgG aCL (188 vs. 10 U/mL; *p* < 0.007), IgG anti-β2GPI (49 vs. 4 U/mL, *p* < 0.003) and IgA anti-β2GPI (15 vs. 7 U/mL; *p* < 0.005), compared with women who did not suffer late miscarriage (Supplementary Figures 1B–D).

### aPL Levels Over Time in Relation to Received Pharmacotherapy

To assess the impact of administered pharmacotherapy on aPL levels, the patients were categorized into different groups. Those who received continuous HCQ therapy showed significantly higher median levels of IgM anti-β2GPI (7 vs. 3 U/mL; *p* < 0.0001) and IgM aCL (11 vs. 5 U/mL; *p* < 0.02) compared with the group that received another Disease-Modifying Anti-Rheumatic Drug (DMARD) or no DMARD (Supplementary Figures 1E,F).

No significant association was found regarding the levels of any aPL isotype and the prescribed daily dose of prednisolone.

### aPL Levels in Relation to Clinical Phenotypes (Classification Criteria)

When using the aPL results as categorical variables, an inverse association between IgG anti-β2GPI and photosensitivity (*p* < 0.008) was observed. In line with this finding, a positive LA test was inversely associated with photosensitivity (*p* < 0.05). IgA anti-β2GPI levels were significantly associated with leukopenia (*p* < 0.04), and IgM anti-β2GPI levels were inversely associated with the presence of Raynaud's phenomenon (*p* < 0.05). Finally, the levels of IgM aCL were significantly associated with hypocomplementemia (low C3 and/or C4) (*p* < 0.03).

### aPL Levels in Relation to Gender, Smoking Habits, and Organ Damage

No significant associations regarding aCL, anti-β2GPI and LA were observed in relation to gender. Similarly, smoking habits did not show any significant associations with aPL levels. However, the presence of IgG anti-β2GPI at any time-point during the follow-up period was significantly associated with severe damage accrual (SDI score ≥4; *p* < 0.03).

## Discussion

The main goal of this prospective study was to evaluate the occurrence and variations of aPL over time among individuals who were recently diagnosed with SLE. Longitudinal studies of aPL are uncommon and most of them have had a specific focus, such as investigating aPL levels from conception and onwards in relation to adverse pregnancy outcomes (27, 33, 34). Depending on which cut-offs that were applied, our data demonstrate a prevalence for aPL of 44–61% (any isotype of aCL, anti-β2GPI or LA at least once during follow-up). This broad range of aPL prevalence underlines the importance of choosing a relevant cut-off. Although the Sydney criteria postulate a 99th percentile (or 40 units) cut-off, according to the widely used external control program UK NEQAS many labs seem to use the cut-offs that are recommended by the manufacturer and these are usually lower (normally corresponding to the level slightly above of the 95th percentile) (4). Lower frequencies of aPL in SLE than we achieved here have previously been reported in cross-sectional studies (5). Three to four cases seroconverted in either direction for both aCL and anti-β2GPI, whereas the median aPL levels numerically decreased over time. Although the aPL levels fluctuated over time, the patients tended to remain aPL-positive or aPL-negative and the clinical value of repeated aPL-testing in the absence of thromboembolic events or new symptoms appears to be limited. However, this study was not powered to fully evaluate how the fluctuation of the antibody levels lead to a variation in the risk of clinical events.

We confirm some established relationships, such as the significantly higher aPL levels in patients with certain APS-related events, such as late miscarriage and MI (35). The strongest associations with vascular events were found for the IgG isotype of aCL and anti-β2GPI, which is in line with observations made by others (1, 6, 36). LA positivity was not significantly associated with any clinical outcome, which was unexpected. This may be attributable to the fact that the LA results were retrieved from the medical records and not analyzed continuously at all visits. As only patients initially testing positive for LA were re-tested, we cannot exclude that subjects initially testing negative for LA could have shown a positive LA test later on. In addition, a limited number of vascular events and a rather high prevalence of recorded LA positivity could have contributed to the lack of associations between LA and APS-related events. Furthermore, biased LA results due to ongoing anticoagulation during follow-up could not be excluded. Nevertheless, an observed association, which has been reported previously, was the relationship between hypocomplementemia and IgM aCL, indicating more pronounced activation of the classical pathway in aPL-positive SLE patients compared to those patients without aPL (1, 37, 38).

Regarding aPL isotypes, the most frequently detected aCL isotype “at inclusion” and during follow-up was IgM, and for anti-β2GPI it was IgA. While the overlap between isotypes was substantial (Table 2), the overlap between aCL, anti-β2GPI and LA appeared to be less extensive. For instance, exclusive anti-β2GPI positivity was detected in seven of the 33 aPL-positive patients (21%) using manufacturer's cut-offs. Compared to previous studies of SLE as well as of primary APS, and regardless of cut-off applied, we obtained a higher percentage of IgA anti-β2GPI-positive cases. The reason for this is not obvious. We have previously reported that analyzing IgA aPL in addition to IgG and IgM in patients with SLE has limited clinical value (23). In a Spanish setting, Ruiz-Irastorza et al. investigated the prevalence of IgG/IgM aCL and/or LA among patients with incident SLE and found that 36% were positive (12). During a mean follow-up of almost 10 years, 43% of the patients tested positive for aCL and/or LA at any time-point. Although the follow-up was significantly shorter in our study, similar results were achieved, with ~39% testing positive for aCL and/or LA over time.

Some studies have indicated that patient ethnicity influences both the aPL levels and aPL isotypes. In a North American setting, Caucasian patients with SLE were shown to have higher levels of IgG aPL whereas African-American patients with SLE showed higher levels of IgA aPL (21). In addition, the presence of anti-β2GPI (all three isotypes) was suggested to be more common among Caucasians than African-Americans. In addition, a recent study has concluded that IgA aPL is more common in Sudanese patients with SLE than in Swedish patients with SLE (39). Furthermore, in the Sudanese control group, IgA aPL was more commonly detected than in the Swedish controls (22). This indicates that the results obtained herein, being based mainly on samples from Caucasian individuals, cannot necessarily be extrapolated to other ethnicities.

An additional aim of this study was to evaluate potential associations between aPL levels and the use of pharmacotherapy. In a comparison of patients who received HCQ continuously and patients prescribed other DMARDs or no DMARD, significantly higher IgM aCL and anti-β2GPI levels were found in the HCQ group, whereas no differences were found for the other isotypes (Supplementary Figures 1E,F). Partly in contrast to our findings, other groups have reported decreasing levels of IgG aCL and IgG/IgM anti-β2GPI in SLE subjects who received HCQ therapy compared with those not treated with HCQ (40). Sciascia et al. reported reduced levels of aCL and anti-β2GPI following belimumab therapy, and co-treatment with HCQ was suggested to have an even greater potential to reduce aPL levels than belimumab alone (41, 42). It is important to keep in mind that, although aPL herein were analyzed at the same occasion in stored samples, the clinicians also achieved aPL results as part of clinical routine. The indication for HCQ therapy might thus have been dependent on aPL positivity and the estimated future risk of thrombosis. In addition, we acknowledge that a non-adherence assessment for background therapies was not performed in the present study. It is well-known that adherence to HCQ is far from optimal among patients with SLE (43).

This study has certain limitations. Relatively few patients were included and the number of APS-related events was low. The latter may be attributed to the close monitoring and generally well-controlled patients. As mentioned, the study was underpowered to evaluate how fluctuations of aPL levels could lead to changed risks of clinical events. The results for LA were cross-sectional and based on “ever positivity.” In contrast, the prospective study design, a study population unbiased from prior organ damage at inclusion and the monitoring of patients at a single rheumatology unit constitute major strengths. Furthermore, the fact that all samples were analyzed by an accredited laboratory and at the same occasion, thereby minimizing the inter-assay variation, was advantageous. The study population had good coverage, with very few missing values during the first 36 months. However, over time further cases were lost to follow-up, mainly due to death or migration (missing data are indicated in Figure 1). Finally, all new SLE patients with recent-onset of disease at our unit during the study period were included, excluding the risk of selection bias.

## Conclusions

This study demonstrates that the presence of aPL in patients with SLE is common at disease onset, and that the percentage of aPL-positive cases increases slightly over time, which is similar to what has been observed in primary APS (28). Previously reported associations between increased levels of IgG aCL and MI, as well as with late miscarriage were confirmed in the present study. In addition, the IgG and IgA anti-β2GPI levels were significantly higher among females who suffered a late miscarriage. Although the aPL levels fluctuated over time, the majority of the patients tended to remain aPL-positive or aPL-negative. Based on our data, we conclude that in the absence of new symptoms or before a planned pregnancy, the value of repeated aPL-testing is limited for patients with SLE but larger longitudinal studies are warranted to shed further light upon this issue.

## Data Availability Statement

The original contributions generated for the study are included in the article/Supplementary Material, further inquiries can be directed to the corresponding author.

## Ethics Statement

The studies involving human participants were reviewed and approved by the Regional Ethics Review Board in Linköping (Decision Nr. M75-08/2008). The patients/participants provided their written informed consent to participate in this study.

## Author Contributions

MF and CS: conceptualization and supervision. MF and CD: methodology and project administration. MF and TW: formal analysis, data curation, and visualization. TW: writing—original draft preparation. MF, CD, and CS: writing—review and editing. All authors: validation, investigation, and have read and agreed to the final version of the manuscript.

## Conflict of Interest

The authors declare that the research was conducted in the absence of any commercial or financial relationships that could be construed as a potential conflict of interest.

## Supplementary Material

Supplementary Figure 1 Anti-phospholipid antibody levels in relation to vascular events, pharmacotherapy received and late miscarriages. Statistically significant associations are noted for: **(A)** IgG anti-cardiolipin (aCL) versus (vs.) myocardial infarction; **(B)** IgG aCL vs. late miscarriage; **(C)** IgG anti-β2-glycoprotein-I (anti-β2GPI) vs. late miscarriage; **(D)** IgA anti-β2GPI vs. late miscarriage; **(E)** IgM anti-β2GPI vs. continuous hydroxychloroquine (HCQ) therapy; and **(F)** IgM aCL vs. continuous HCQ therapy.
