# Supplementary figures and images for: Longitudinal Analysis of Anti-cardiolipin and Anti-β2-glycoprotein-I Antibodies in Recent-Onset Systemic Lupus Erythematosus: A Prospective Study in Swedish Patients
Source: Front Med (Lausanne). 2021 Feb 24;8:646846. doi: 10.3389/fmed.2021.646846 (PMC7959716; doi:10.3389/fmed.2021.646846)

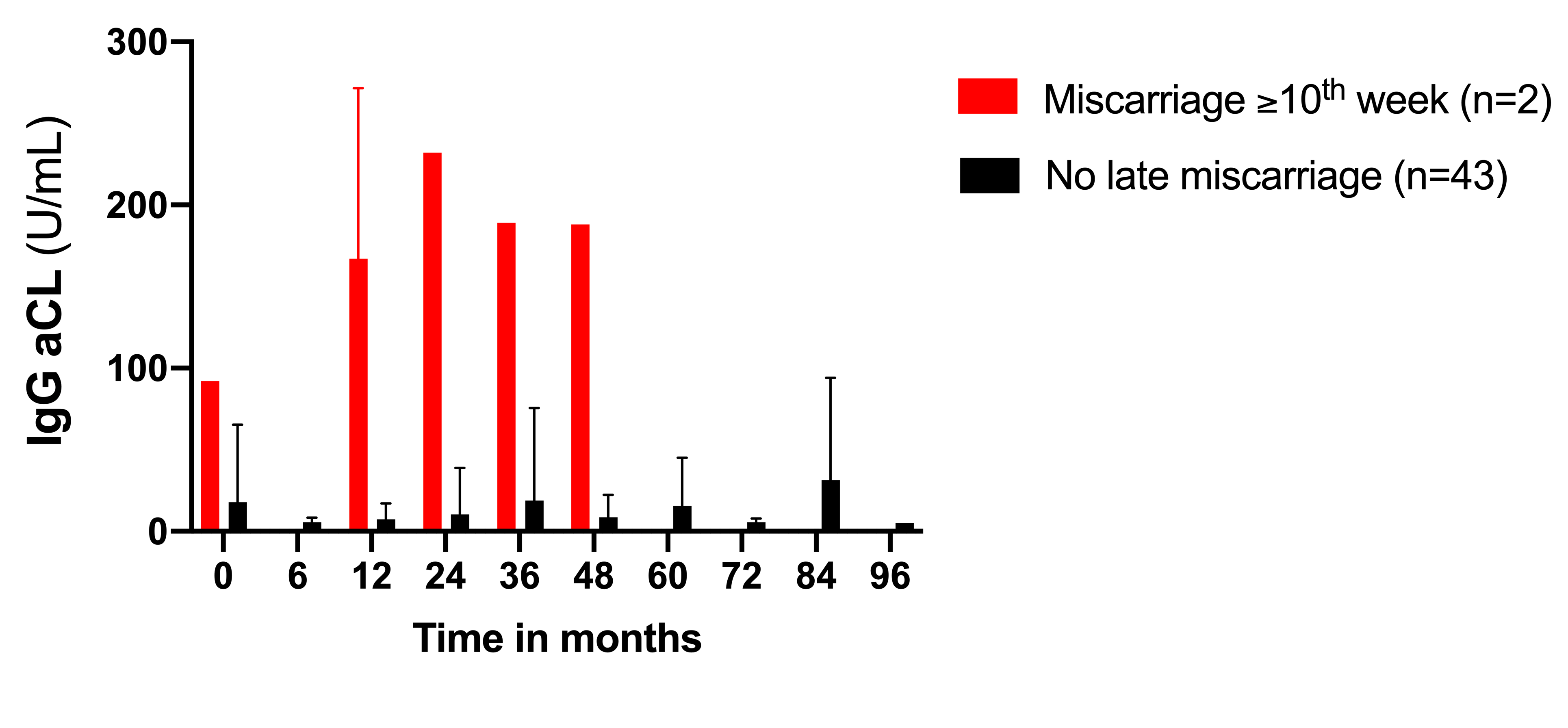

Supplement: Supplementary file 1 [file Image_2.TIFF]

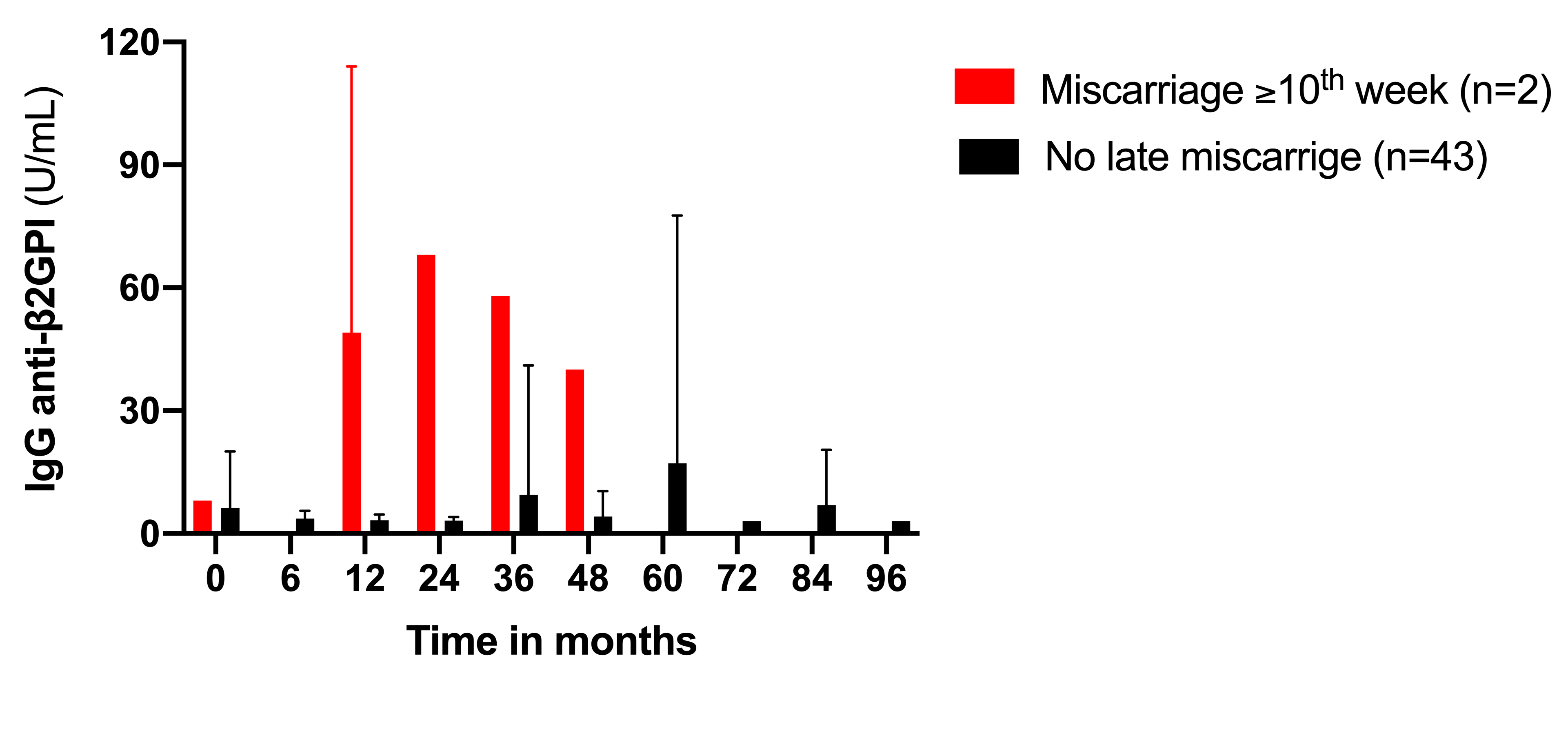

Supplement: Supplementary file 2 [file Image_3.TIFF]

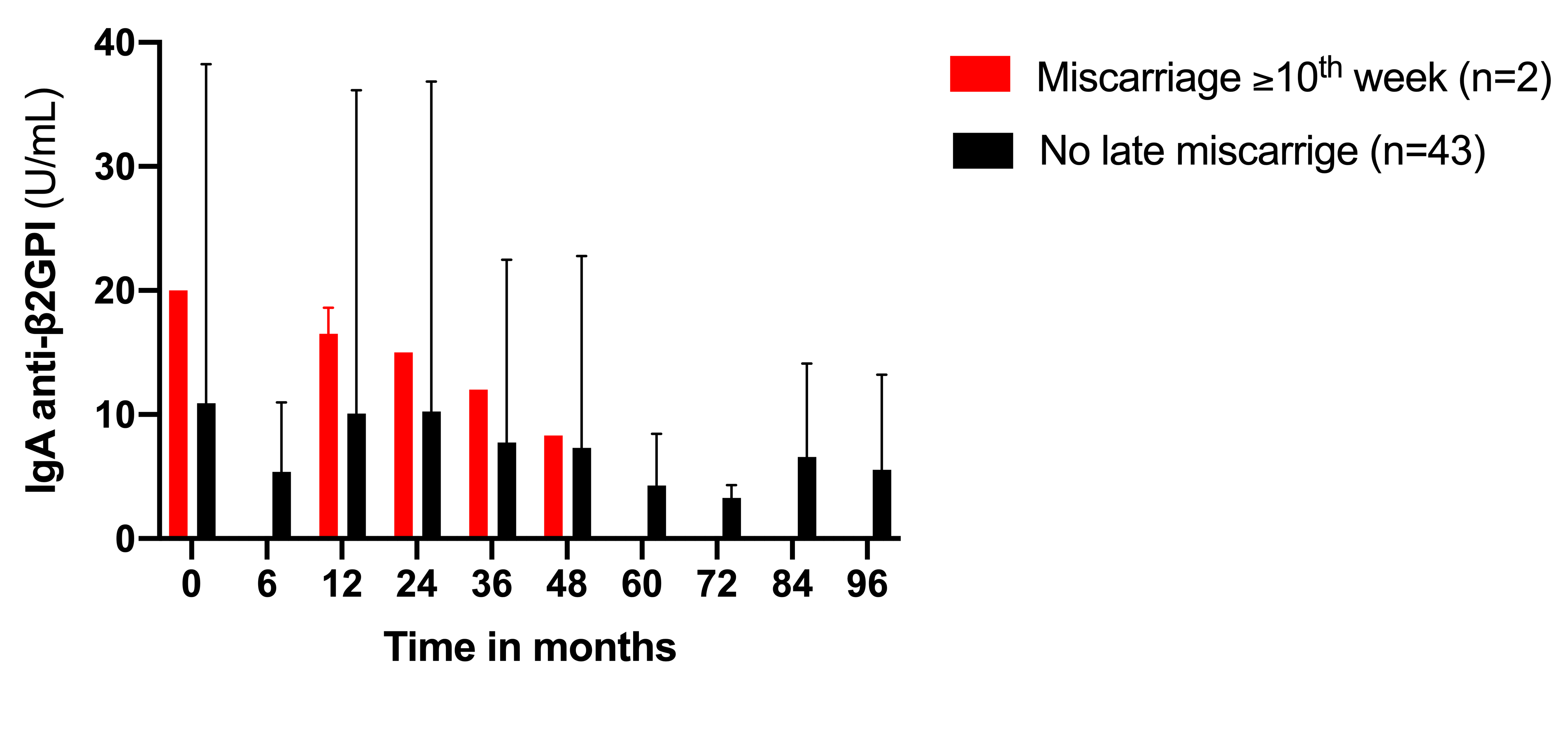

Supplement: Supplementary file 3 [file Image_4.TIFF]

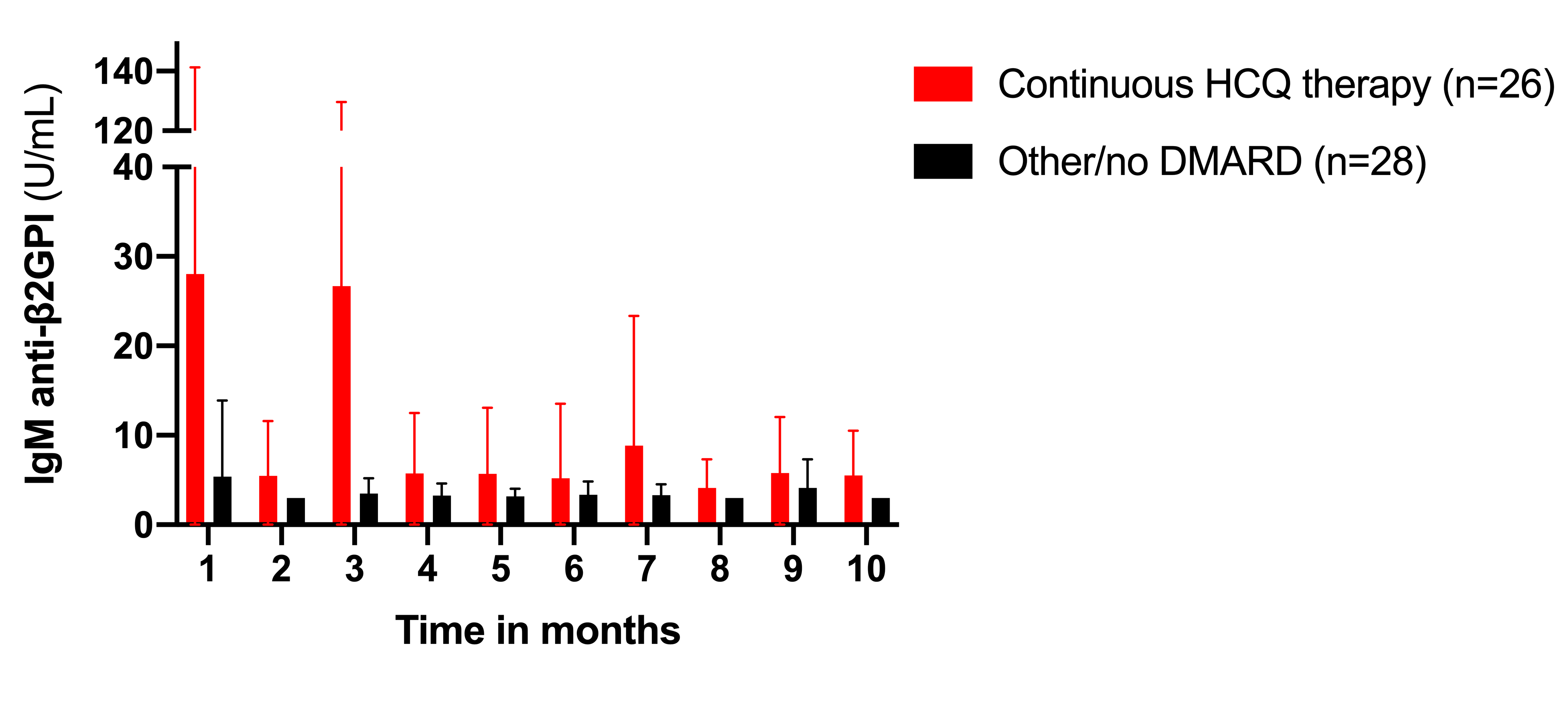

Supplement: Supplementary file 4 [file Image_5.TIFF]

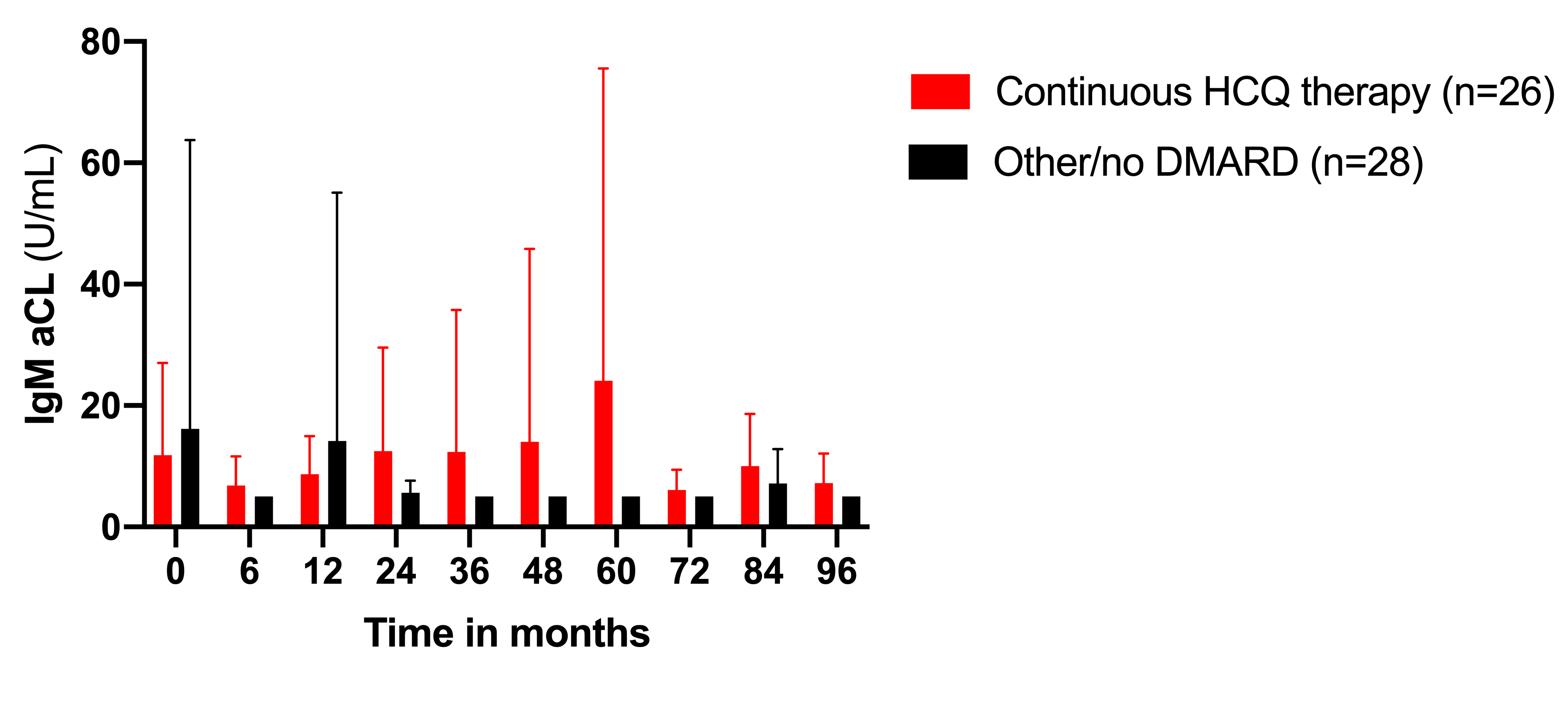

Supplement: Supplementary file 5 [file Image_6.TIFF]

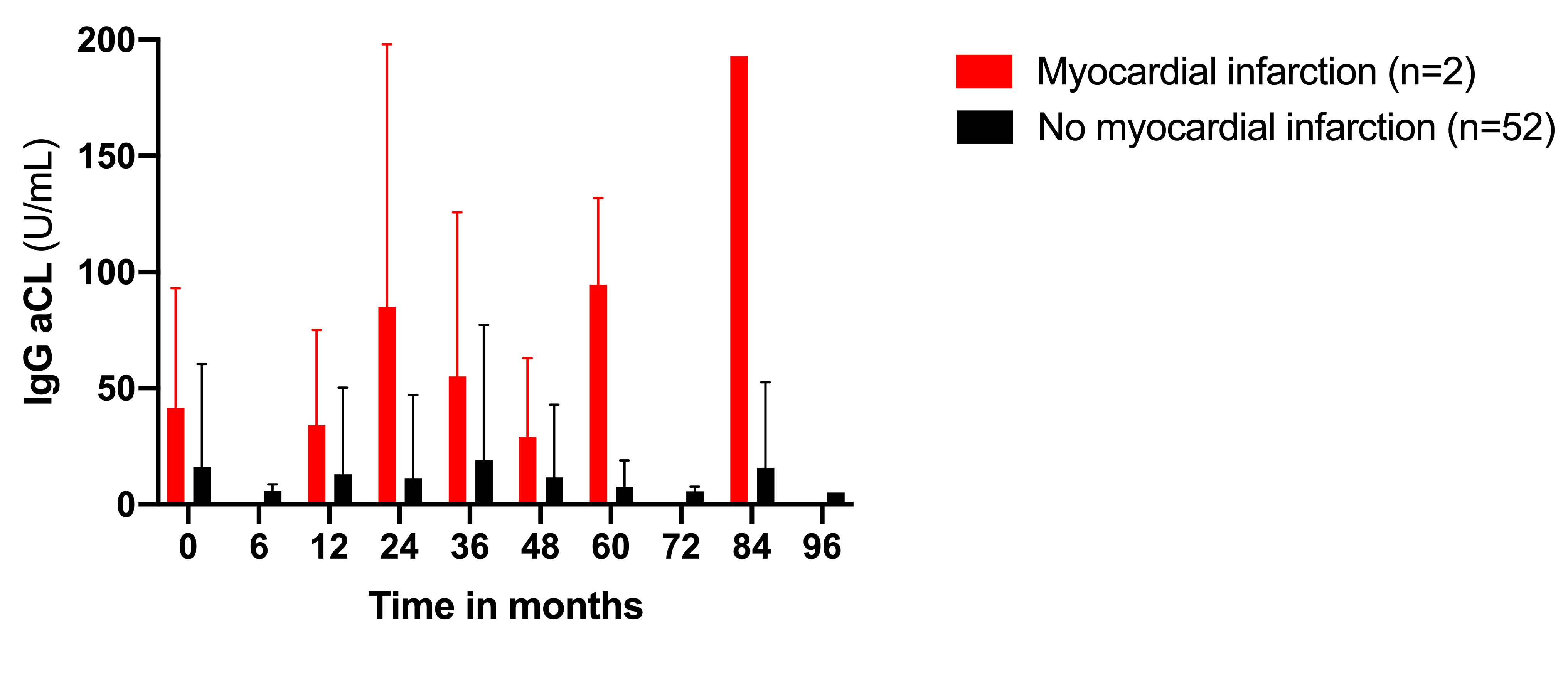

Supplement: Supplementary Figure 1 — Anti-phospholipid antibody levels in relation to vascular events, pharmacotherapy received and late miscarriages. Statistically significant associations are noted for: (A) IgG anti-cardiolipin (aCL) versus (vs.) myocardial infarction; (B) IgG aCL vs. late miscarriage; (C) IgG anti-β2-glycoprotein-I (anti-β2GPI) vs. late miscarriage; (D) IgA anti-β2GPI vs. late miscarriage; (E) IgM anti-β2GPI vs. continuous hydroxychloroquine (HCQ) therapy; and (F) IgM aCL vs. continuous HCQ therapy. [file Image_1.TIFF]
